# Coimmunomodulation of tumor and tumor-draining lymph nodes during in situ vaccination promotes antitumor immunity

**DOI:** 10.1172/jci.insight.146608

**Published:** 2022-06-22

**Authors:** Moonkyoung Jeong, Heegon Kim, Junyong Yoon, Dong-Hyun Kim, Ji-Ho Park

**Affiliations:** 1 Department of Bio and Brain Engineering and KAIST Institute for Health Science and Technology, Korea Advanced Institute of Science and Technology (KAIST), Daejeon, South Korea.; 2 Department of Radiology, Feinberg School of Medicine, Northwestern University, Chicago, Illinois, USA.; 3 Department of Bioengineering, University of Illinois at Chicago, Chicago, Illinois, USA.; 4 Robert H. Lurie Comprehensive Cancer Center, Chicago, Illinois, USA.; 5 Department of Biomedical Engineering, McCormick School of Engineering, Northwestern University, Evanston, Illinois, USA.

**Keywords:** Therapeutics, Vaccines, Cancer, Cancer immunotherapy, Drug therapy

## Abstract

In situ vaccination has demonstrated the feasibility of priming local immunity for systemic antitumor responses. Although direct intratumoral (IT) delivery of adjuvant is the mainstay, tumor-draining lymph nodes (TDLNs) also play essential roles in antitumor immunity. We report that directing an adjuvant to both tumors and TDLNs during in situ vaccination can induce robust antitumor responses. Conventional IT dosing leads to tumor-limited delivery of agents; however, delivery to both tumors and TDLNs can be ensured through a micellar formation. The peritumoral delivery of micellar MEDI9197 (mcMEDI), a toll-like receptor 7/8 agonist, induced significantly stronger innate and adaptive immune responses than those on conventional dosing. Optimal dosing was crucial because excessive or insufficient accumulation of the adjuvant in the TDLNs compromised therapeutic efficacy. The combination of local mcMEDI therapy significantly improved the efficacy of systemic anti–programmed death receptor 1 therapy. These data suggest that rerouting adjuvants to tumors and TDLNs can augment the therapeutic efficacy of in situ vaccination.

## Introduction

Cancer immunotherapy has shown significant advances over the past decade with the success of immune checkpoint blockades (ICBs) directed against programmed death receptor 1 (PD-1) and cytotoxic T-lymphocyte–associated protein 4 (CTLA-4; refs. 1, 2). However, the benefits of ICBs are restricted to a minority of patients and their combination can cause severe immune-related adverse events. Therefore, the development of treatment modalities that can be safely interdigitated with ICBs will improve their efficacy and broaden the clinical applications of immunotherapy.

Directly administering immunomodulatory agents into tumors, i.e., in situ vaccination, has demonstrated the feasibility of priming local immunity for systemic antitumor responses (3–6). Immunotherapy can be tremendously improved by inducing the superior priming of antitumor responses locally and reducing off-target toxicities. In addition, this modality has broad applicability because the tumor antigens need not be identified. There are many ongoing clinical trials on intratumoral (IT) immunotherapy as monotherapy or combination therapy with conventional treatments in varying types and at different stages of cancer (7, 8). Efforts to integrate immuno-oncology into interventional oncology will broaden the applicability of local modalities for various lesions within the body (9).

TLR agonists are small molecules that activate the innate immune system and are one of the most utilized immune stimulators under active investigation preclinically (3, 4) and clinically (10, 11) for in situ vaccination. However, they are not completely free from safety issues because intratumorally injected small molecules tend to rapidly disseminate into the systemic circulation and can cause systemic adverse events (12–15). They require not only systemic toxicity evaluation but also strategies for local distribution modulation. MEDI9197, a lipophilic TLR 7/8 agonist with a lipid tail, was designed to improve IT dosing by enhancing its retention within the tumor and minimizing adverse events, including systemic exposure (4). It induces both systemic and IT immune responses in patients with solid tumors, implying its potential in combination immunotherapies (NCT02556463; ref. 16).

Tumor-draining lymph nodes (TDLNs) receive lymphatic drainage from tumors and play essential roles in initiating and regulating antitumor immunity (17–19). TDLN-targeted immunotherapies have been known to be effective in inducing antitumor responses (20–24). Even though IT administration is the mainstay, whether this route of administration is optimum for maximizing TDLN modulation is still unclear. Furthermore, there have been few attempts to modulate both tumors and TDLNs to evaluate the therapeutic implications of developing such strategies.

We hypothesized that conventional IT dosing did not fully exploit the therapeutic potential of TDLNs and sought to take advantage of the antitumor functions of TDLNs in the course of in situ vaccination. A peritumoral (PT) injection of a micellar formulation of MEDI9197 significantly improved antitumor immune responses compared with that after an IT injection of a conventional formulation, by directing the injected agent efficiently to both tumors and TDLNs. A majority of in situ vaccinations focus on modulating the tumor microenvironment; however, strategies to actively involve TDLNs may provide promising approaches to improve cancer immunotherapy.

## Results

### Efficient delivery to both tumors and TDLNs using a micellar formulation.

Local administration via either the PT or IT route has been previously exploited for in situ vaccination (3, 20). Following local administration, agents with a diameter of 10–100 nm efficiently travel within the tissue and drain into the local lymphatic system (20, 25–27). In addition, proper surface engineering with soluble polymers, such as PEG, is required to facilitate their clearance from the injection site and efficient transport through the interstitial matrix (27, 28). Thus, the feasibility of local administration of PEGylated micelle formulations to tumors and TDLNs was assessed. PEGylated phospholipid, 1,2-distearoyl-sn-glycero-3-phosphoethanolamine-N-[methoxy(polyethylene glycol)-2000] (DSPE-PEG-2000), which is certified by the United States FDA for clinical use, was used for micelle preparation (29, 30). The fluorophore, 1,1¢-dioctadecyl-3,3,3¢,3¢-tetramethylindotricarbocyanine iodide (DiR) was incorporated into the core of the micelles as a model drug. A simple film hydration method was used to produce micelles which were 17.1 ± 2 nm in diameter, with a surface charge of –3.5 ± 0.7 mV (Supplemental Figure 1, A and B, and Supplemental Table 1; supplemental material available online with this article; https://doi.org/10.1172/jci.insight.146608DS1).

**Figure 1:**
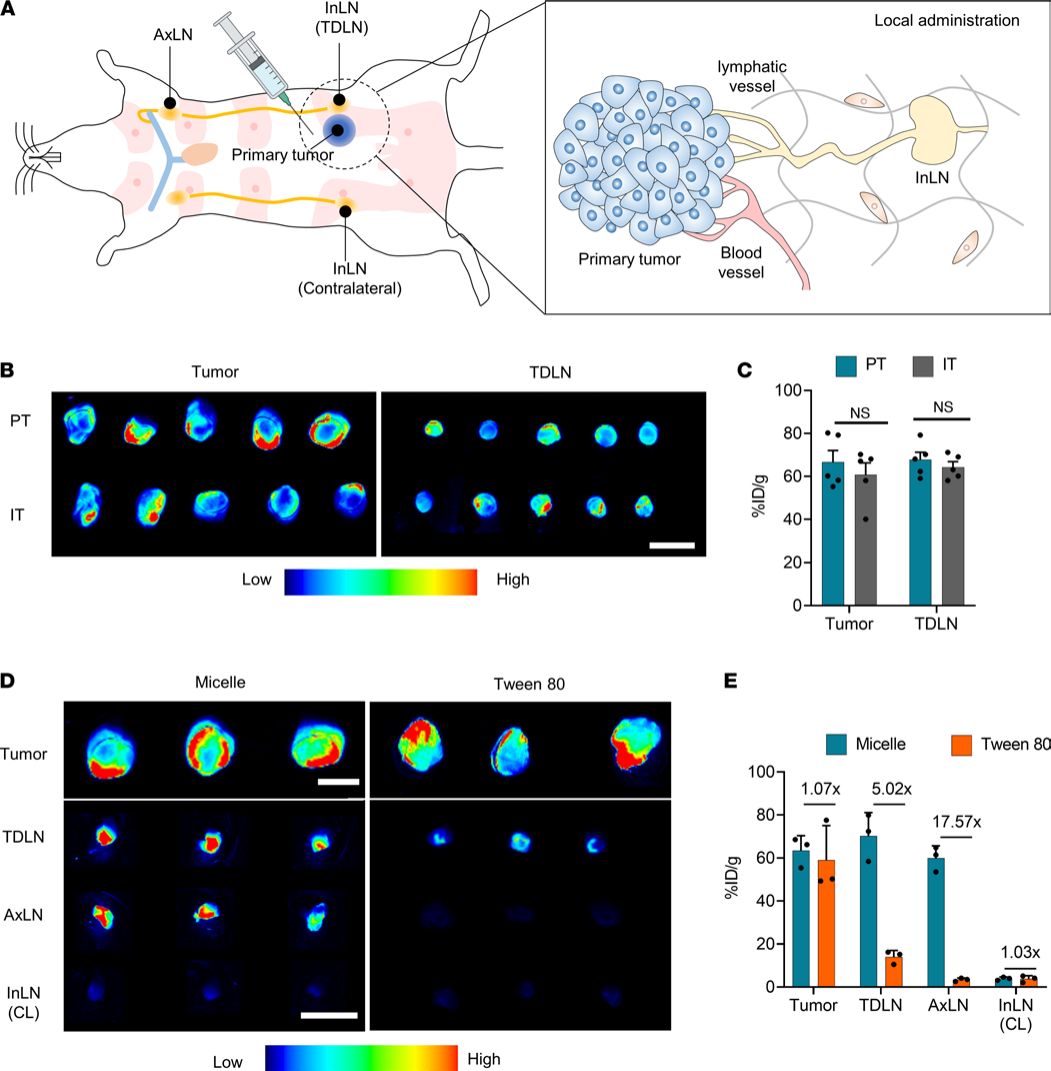
Local administration of micelles facilitates primary tumor and TDLN delivery. BALB/c mice were injected with 2×10^5^ 4T1-Luc mammary carcinoma cells into the fourth mammary fat pad. When the tumor size reached approximately 50 mm^3^, micelles incorporating DiR were administered locally. (**A**) Schematic of local administration. Peritumoral administration was performed by injecting 50 μL of solutions into the peritumoral region approximately 5 mm away from the tumor. IT administration was performed by injecting 10 μL of solutions directly into the center of the tumor. (**B** and **C**) Ex vivo fluorescence images of tumor and TDLNs at 96 hours after peritumoral or IT micelle dosing and their quantification (*n* = 5). (**D** and **E**) Either conventional IT dosing of DiR or peritumoral dosing of micelle incorporating DiR was performed in 4T1-Luc tumor-bearing mice. For conventional IT dosing, DiR was first dissolved in Tween 80 (40 mg/mL) and then ethanol (2.5 mg/mL) sequentially, which was diluted with PBS into a final concentration. IT administration was performed by injecting 10 μL of solutions directly into the center of the tumor. Ex vivo fluorescence images of tumor and TDLNs at 96 hours after micelle or conventional IT dosing shown in D and their quantification in E (*n* = 3). Scale bars: 5 mm. Unpaired 2-tailed Student’s *t* test. Data are representative of 2 independent experiments. Data presented as mean ± SEM. LN, lymph node; In, inguinal; Ax, axillary; CL, contralateral; %ID/g, percent of injected dose per gram tissue.

The more effective route of administration of delivering drugs to both tumors and TDLNs was investigated. For local administration, an orthotopic breast tumor model was established by implanting 4T1-Luc mammary carcinoma cells into the fourth mammary fat pad of mice, where the inguinal lymph node (InLN) is located and serves as a TDLN. PT and IT were administered by injecting 50 μL of micelles into the PT region, approximately 5 mm away from the tumor, and 10 μL directly into the center of the tumor (Figure 1A). As a result, PT and IT administration exhibited comparable tumor and TDLN delivery efficiency (Figure 1, B and C), showing that both routes can be exploited interchangeably using the micelle formulation. Thereafter, the PT route for micelle administration was exploited, because it did not induce backflow of the injected solution or bleeding, both of which are often observed after IT administration. The micelle formulation was compared with a conventional IT formulation prepared using Tween 80, commonly used for the delivery of lipophilic drugs (31, 32). While the micelle and conventional formulations had comparable tumor delivery efficiency, the micelle formulation achieved superior delivery to the TDLN (Figure 1, D and E), implying the limited effect of conventional IT dosing on directing the injected agents to TDLNs. Micelles reached peak concentration in blood before 24 hours after injection, and less than 1% remained in the blood at 96 hours following injection (Supplemental Figure 1C). The distribution of micelles in the major organs at 96 hours following injection showed no significant differences between the 2 formulations (Supplemental Figure 1D), suggesting that their systemic exposures were similar. Taken together, these results suggest that the micellar formulation can facilitate delivery to the tumor and TDLN via local administration.

### Preparation and characterization of micellar MEDI9197.

**Figure 2:**
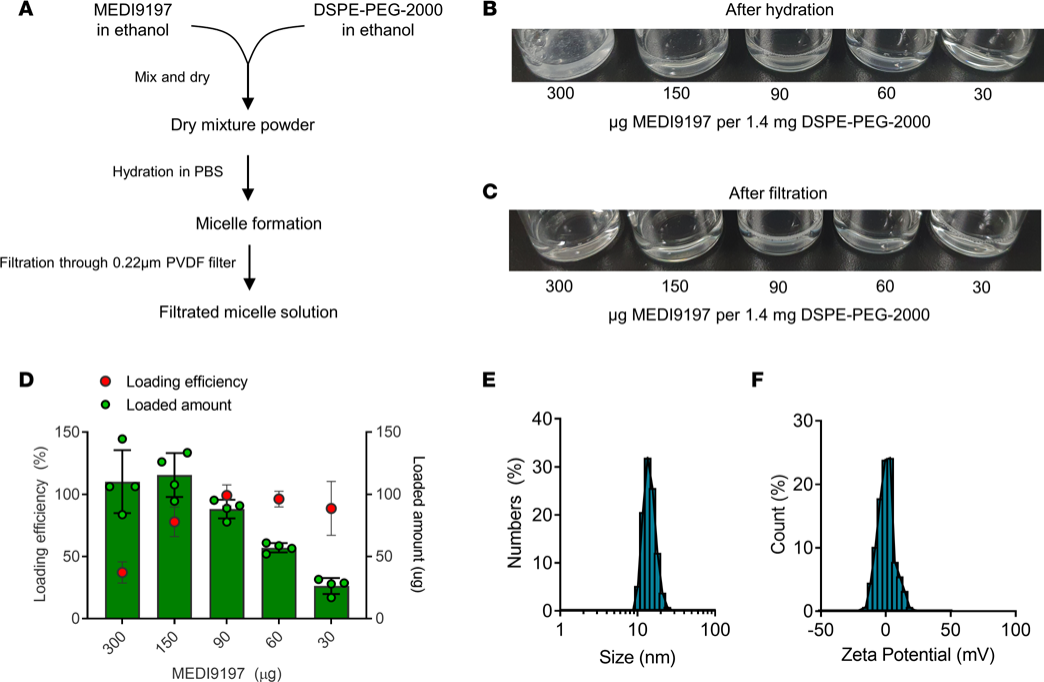
Development and characterization of micellar MEDI9197. (**A**) Schematic of diagram of micellar MEDI9197 preparation. (**B**) A representative image of dried film composed of MEDI9197 and DPSE-PEG-2000. (**C**) Representative images of solutions containing micellar MEDI9197 before and after filtration. The numbers indicate molar ratio of MEDI9197 to DSPE-PEG-2000. (**D**) Loading efficacy of MEDI9197 at various molar ratios of MEDI9197 to DSPE-PEG-2000 (*n* = 4). (**E** and **F**) Representative distribution profiles of hydrodynamic diameter and zeta potential distribution. Data are representative of 3 independent experiments. Data presented as mean ± SEM.

The lipophilicity of MEDI9197 for micellar incorporation was utilized (33). A simple film hydration method was used for formulating micelles loaded with MEDI9197, followed by a filtration process to remove the unloaded MEDI9197 (Figure 2A). To assess the loading capacity and efficiency of micelles, varying doses of MEDI9197 were used, ranging from 30 μg to 300 μg per 1.4 mg DSPE-PEG-2000. Hydration of the film resulted in a transparent solution, except at the highest MEDI9197 dose, in which the solution had visible aggregates, indicating that the dose exceeded the capacity of micelles to load MEDI9197 (Figure 2B). The solution was filtrated through a 0.2 μm membrane pore filter, and the insoluble MEDI9197 was removed (Figure 2C). It was found that 90 μg of MEDI9197 could be loaded into the PEGylated micelles with almost no loss (> 99% loading efficiency) (Figure 2D), as measured by the UV spectrum of MEDI9197 (Supplemental Figure 2). In the experiments that followed, this micellar formulation called mcMEDI was used. mcMEDI was 14.3 ± 1.7 nm in diameter, with a surface charge of –1.8 ± 0.5 mV (Figure 2, E and F, and Supplemental Table 1).

### Local administration of mcMEDI for immunomodulation of tumors and TDLNs.

**Figure 3:**
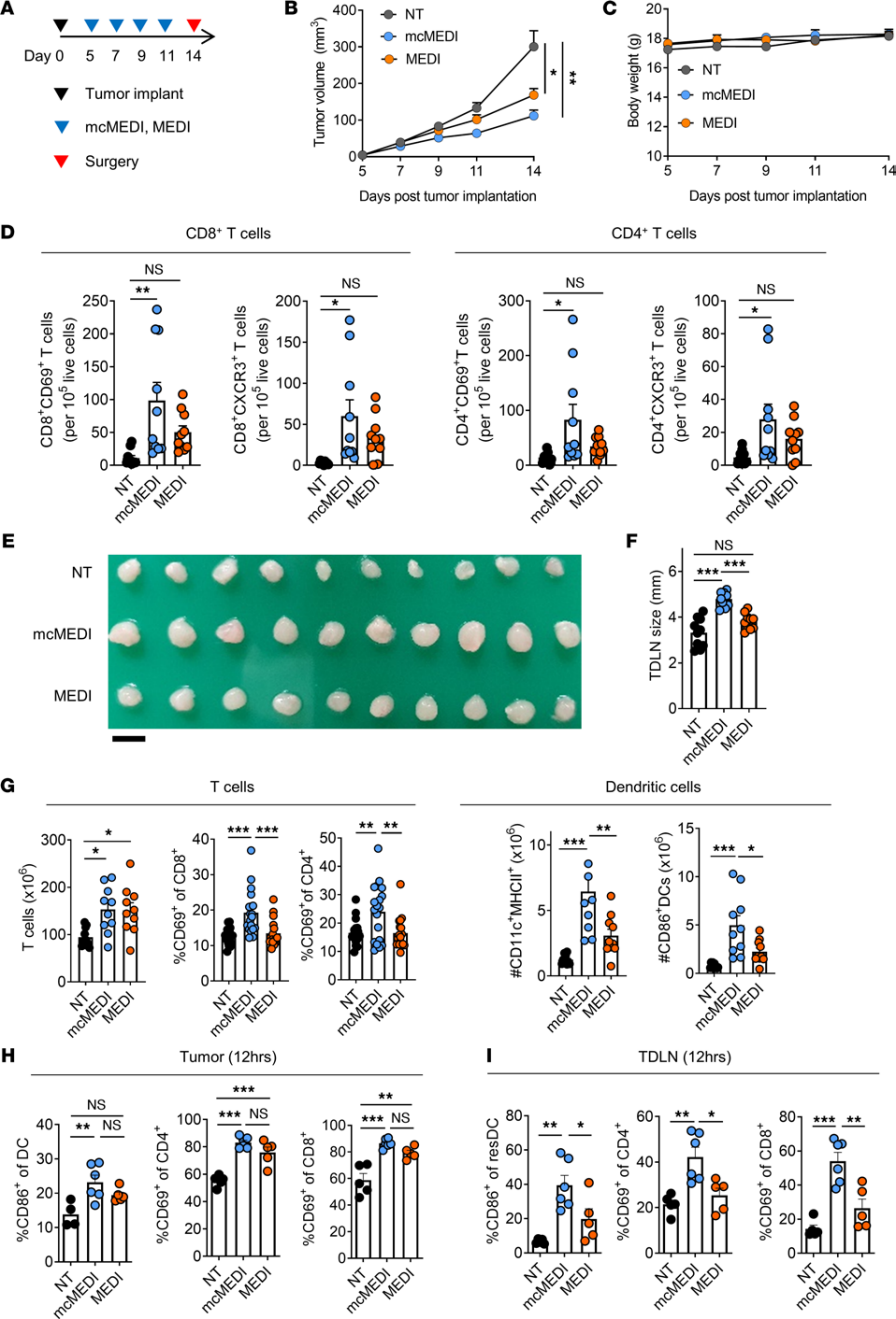
mcMEDI therapy induces effective local immune activity within primary tumor and TDLN. (**A**) Schematic of treatment plan. BALB/c mice (*n* = 10 per group) were injected with 2 × 10^5^ 4T1-Luc mammary carcinoma cells into the fourth mammary fat pad (day 0). Mice were injected peritumorally with 50 μL of mcMEDI and were injected intratumorally with 10 μL of MEDI9197. Both treatment groups were given MEDI9197 at a dose of 0.5 μg per injection at the indicated days. The NT group did not receive MEDI9197. Primary tumors and TDLNs were dissected 14 days after implantation and digested to isolate single cells for the flow cytometry analysis. (**B**) Primary tumor growth and (**C**) BW of mice during therapy. (**D**) Activated (CD69^+^) and effector (CXCR3^+^) T cell phenotypes (CD8^+^ or CD4^+^) in individual tumors per 10^5^ live cells are shown. (**E**) Ex vivo images of dissected TDLNs and (**F**) their size. Scale bar: 5 mm. (**G**) Activated (CD69^+^) T cells, activated (MHCII^+^, CD86^+^) DCs in individual TDLNs are shown. (**H** and **I**) 4T1 tumor-bearing BALB/c mice (*n* = 5–6 per group) were injected with 0.5 μg of mcMEDI (PT) or MEDI9197 (IT) once at day 7. Dissected tumors and TDLNs at 12 hours after injection were digested into single cells, stained, and assessed by flow cytometry. H shows the frequency of activated dendritic cells (CD86^+^) or T cells (CD69^+^) in tumor tissue, and I shows the frequency of activated resident DCs (CD86^+^) and T cells (CD69^+^) in TDLNs. Data are representative of 3 independent experiments for **A–G** and 2 independent experiments for **H** and **I**. Data presented as mean ± SEM. *P < 0.05, **P < 0.01, ***P < 0.001; 2-way RM ANOVA and Tukey’s multiple comparisons test for **B** and **C**, 1-way ANOVA and Tukey’s multiple comparisons test for D and F–I.

The induction of immune responses in both tumors and TDLNs following the PT injection of mcMEDI was assessed. Mice were implanted with 4T1-Luc mammary carcinoma cells orthotopically into their fourth mammary fat pad. Then, 50 μL of mcMEDI solution was injected into the PT region at a distance of approximately 5 mm from the tumor (mcMEDI group). For conventional IT dosing, 10 μL of MEDI9197 prepared using Tween 80 was directly injected into the center of the tumor (MEDI group). The dose of MEDI9197 for the groups was 500 ng per injection, and the treatment was initiated 5 days after implantation, followed by 3 more injections every other day. Tumors and TDLNs were dissected 14 days following implantation and analyzed for their immune profile (Figure 3A). The peritumoral dosing of bare micelle solution and the IT dosing of free MEDI9197 excipient (PBS containing 0.125% Tween 80 and 2% ethanol) did not show a significant difference in primary tumor volume (Supplemental Figure 3A) or TDLN size (Supplemental Figure 3B) compared with the no treatment (NT) group. mcMEDI inhibited tumor growth most effectively as measured by tumor volume (Figure 3B and Supplemental Figure 3C) and weight (Supplemental Figure 3D), and there was no significant difference in BW between the groups during the treatment (Figure 3C). When the immune-related pathologic responses induced by MEDI9197, which featured an IT influx of activated T cells (4), were assessed, the mcMEDI group exhibited significantly increased frequencies of both CD8^+^ and CD4^+^ T cells in their activation (CD69^+^) or effector (CXCR3^+^) status. The MEDI group showed no significant increase in the population compared with that in the NT group (Supplemental Figure 4 and Figure 3D). IT T cell phenotypes showed robust negative correlation with tumor burden (Supplemental Figure 5). CD4^+^ T cells, especially the CD4^+^CXCR3^+^ phenotype, exhibited a strong positive correlation with that of CD8^+^ T cells and their effector phenotypes (Supplemental Figure 6), implying the involvement of both cell types and their interplay in effective immune responses (34, 35). When examining the dissected TDLNs, it was found that the TDLNs in the mcMEDI group exhibited significantly greater enlargement than those in the NT and MEDI groups, indicating lymphocyte proliferation (Figure 3, E and F). Flow cytometry analysis revealed that the TDLNs in the mcMEDI group exhibited a significantly greater increase in the activation of T cells and DCs (MHCII^+^, CD86^+^) than those in the MEDI group (Figure 3G and Supplemental Figure 7). Collectively, these data demonstrate that the PT injection of mcMEDI improved local immune responses for antitumor immunity in TDLNs while exhibiting immunomodulation of tumors comparable to IT administration of MEDI9197.

To investigate whether the efficient delivery of mcMEDI to tumors and TDLNs directly modulates the local immune system, immune profiling at an earlier time after injection was performed. At 12 hours after injection, which is the time point before DC migration to TDLNs or MEDI9197-mediated tumor apoptosis occurred, the mcMEDI group showed significantly increased frequencies of activated DCs (CD86^+^) and T cell subpopulations (CD69^+^) in the tumor tissue (Figure 3H). However, the tumor immune profiling was similar between the mcMEDI and MEDI groups, indicating that both treatments were sufficiently capable of activating tumor-resident antigen-presenting cells to provoke antitumor immunity. In contrast, the mcMEDI group exhibited a significant increase in the frequencies of activated resident DCs and T cell subpopulations in the TDLNs compared with those in the MEDI group (Figure 3I). These results suggest that mcMEDI not only modulates TDLNs through tumor-to-TDLN migration of activated DCs but also directly activates resident DCs in the TDLN through lymphatic drainage. Due to this combination effect, peritumorally injected mcMEDI exhibited superior efficacy in modulating the TDLN than intratumorally injected MEDI9197 (Figure 3G).

It was also investigated whether the PT injection of mcMEDI induces the local immunomodulation in a mouse melanoma model. MO5 (OVA-expressing B16F10) melanoma cells were s.c. injected into the flanks of the mice. mcMEDI or MEDI9197 was administered as in the case of the 4T1 model. The treatment was initiated 10 days after implantation, followed by 3 more injections every other day. TDLNs were dissected 19 days following implantation and analyzed for immune profiling. As observed in the 4T1 model, even though there was only a negligible difference in tumor volume, the mcMEDI group showed superior immunomodulation on the TDLNs compared with that by the MEDI group (Supplemental Figure 8). These results suggest that the PT injection of mcMEDI shows effective immunomodulation of tumors and TDLNs in various types of solid tumors.

### In situ vaccination with mcMEDI for systemic and long-term antitumor immunity.

**Figure 4:**
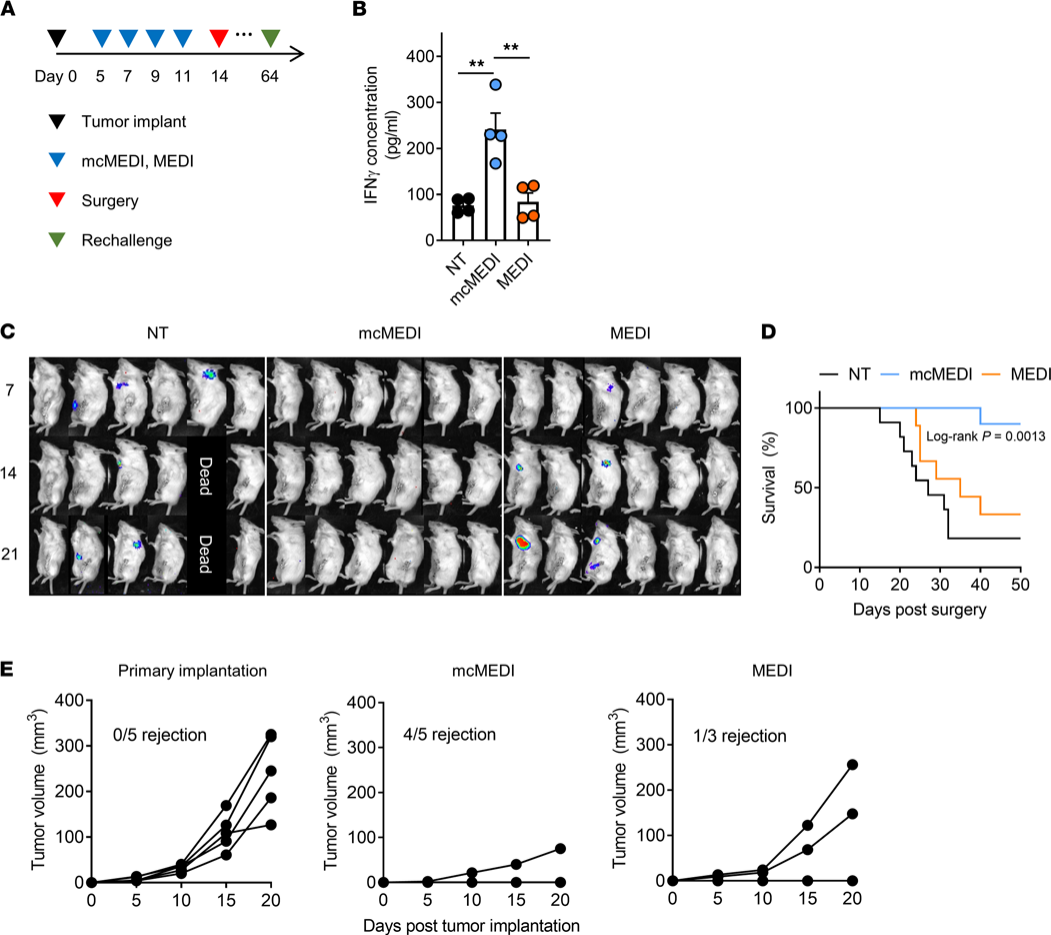
mcMEDI therapy promotes systemic and long-term antitumor immunity. (**A**) Schematic of treatment plan. The injections were administered as was done for Figure 3. At day 14, the primary tumors and TDLNs were dissected with the complete removal of mammary fat tissue and tumor-overlying skin. At day 18, 4 mice from each group were euthanized for splenocytes restimulation while the others were observed until day 64 for survival analysis. (**B**) Splenocytes from each group were cultured for 96 hours in the presence of a specific immunodominant peptide expressed by 4T1 cells (gp70_423–431_). Enzyme-linked immunosorbent assay was performed for quantification of IFN-γ secreted by the splenocytes (*n* = 4 per group). (**C**) Whole body in vivo bioluminescence imaging of 4T1-Luc cells in mice after surgery (*n* = 6 per group). (**D**) Kaplan-Meier survival curves of mice (*n* = 9–11 per group). (**E**) Tumor growth curve after tumor rechallenge. For tumor rechallenge, 1 × 10^4^ 4T1-Luc cells were inoculated in the contralateral mammary fat pad of mice that survived for 50 days in the mcMEDI/MEDI group or naive mice of similar age. Data are representative of 2 independent experiments for A–D. The experiment for E was performed once. Data presented as mean ± SEM. **P < 0.01; 1-way ANOVA and Tukey’s multiple comparisons test for B, log-rank (Mantel-cox) test for D.

It was then investigated whether in situ vaccination with mcMEDI forms systemic adaptive immunity against cancer. Mice implanted with 4T1-Luc mammary carcinoma cells were treated with either mcMEDI or MEDI. The dose of MEDI9197 for the groups was 500 ng per injection and the treatment was initiated 5 days after implantation, followed by 3 more injections every other day. Tumors and TDLNs were surgically removed 14 days following implantation. To minimize the primary tumor recurrence at the surgery site, the skin directly overlying the tumor as well as mammary fat tissue were also removed along with the tumor and TDLNs. The mice were either sacrificed 4 days after surgery for spleen isolation or imaged for the luminescence of 4T1-Luc cells 50 days after surgery to observe for tumor recurrence and survival (Figure 4A). When the splenocytes, isolated from mice, were incubated with gp70_423-431_, an immunodominant peptide expressed by 4T1 cells (36), the mcMEDI group exhibited significantly higher IFN-γ production compared with that in the other groups (Figure 4B). Such efficacy did not accompany a significant increase in proinflammatory cytokine levels, including TNF-α and IFN-γ, in the blood, implying a low risk for systemic immune-related adverse events (Supplemental Figure 9). To investigate the successful formation of systemic adaptive immunity against cancer, 4T1-Luc cells were viewed using an in vivo imaging system (IVIS) after primary tumor surgery. Local recurrences at the surgical sites were observed in both NT and MEDI groups along with lung metastasis, presumably due to the incomplete removal of primary tumors or the local/systemic inflammation originating from perioperative events (37). Nevertheless, the mcMEDI group showed less tumor recurrence, indicating superior systemic immune responses in the mcMEDI group than the MEDI group (Figure 4C). However, about 20% of mice from the NT group did not show tumor recurrence and survived until the end of the observation. To further observe the clear difference in lung metastasis between the groups, the tumor, TDLN, and fat tissue except skin were surgically removed 14 days after implantation to avoid mice without tumor recurrence, and the extent of lung metastasis 21 days after surgery was assessed. The total luminescence radiance of the lung was measured via IVIS and showed a significant decrease in metastasis in the mcMEDI group, despite the remnant tumor intensity being similar between the groups at 7 days after surgery (Supplemental Figure 10). Consequently, the mcMEDI group showed a significant survival benefit (Figure 4D). To assess long-term memory effects, the mice from the mcMEDI and MEDI groups that survived for 50 days following surgery were rechallenged with 4T1-Luc cells in the contralateral mammary fat pad. As a result, 80% of mice in the mcMEDI group and 33% in the MEDI group rejected the tumor, whereas none of the mice in the primary implantation group showed rejection (Figure 4E). Overall, these results suggest that in situ vaccination with mcMEDI can safely augment systemic and long-term antitumor immunity by facilitating the delivery of adjuvant to both tumors and TDLNs.

### Optimization of a mcMEDI dose for effective systemic antitumor immunity.

**Figure 5:**
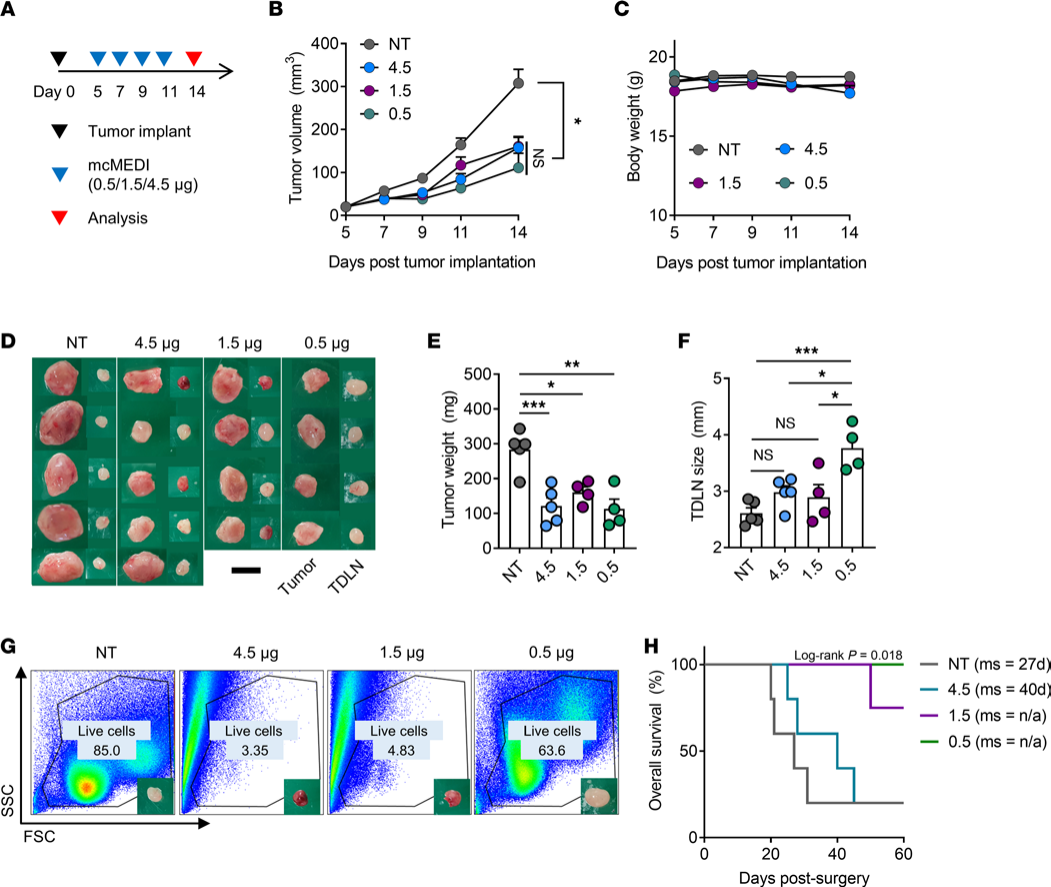
Optimal dose of mcMEDI is required for effective systemic antitumor immunity. (**A**) Schematic of treatment plan. BALB/c mice (*n* = 4–5 per group) were injected with 2 × 10^5^ 4T1-Luc mammary carcinoma cells into the fourth mammary fat pad (day 0). Treatment groups were given mcMEDI peritumorally with various doses of 4.5 μg, 1.5 μg, and 0.5 μg per injection at days 5, 7, 9, and 11. The NT group did not receive MEDI9197. Tumors and TDLNs were dissected at day 14. (**B**) Primary tumor growth and (**C**) BW of mice during therapy. (**D**) Ex vivo images of the primary tumor and TDLNs surgically removed from mice. Scale bar: 5 mm. (**E** and **F**) Weight of tumor and size of TDLN. (**G**) Representative flow dot plots showing TDLN cell loss in high dose regimens. (**H)** Kaplan-Meier survival curves of mice. Median survival days (ms) are listed. The experiment was performed once. Data presented as mean ± SEM. *P < 0.05, **P < 0.01, ***P < 0.001; 2-way RM ANOVA and Tukey’s multiple comparisons test for B and C, 1-way ANOVA and Tukey’s multiple comparisons test for D and E, log-rank (Mantel-Cox) test for H. n/a, not achieved.

Establishing local dosing is essential to exert optimum immunomodulation and maximize adaptive immune responses (38–40). A recent preclinical study demonstrated that excessive and cytotoxic dosing of stimulator of interferon genes (STING) agonists hampers tumor-specific T cell expansion and compromises durable antitumor immunity (41). To assess whether dose escalation can lead to enhanced antitumor immunity, the therapeutic efficacies of mcMEDI at varying doses of 0.5, 1.5, and 4.5 μg per injection were compared. 4T1-Luc–bearing mice were given mcMEDI peritumorally at varying doses of 0.5 μg, 1.5 μg, and 4.5 μg per injection on days 5, 7, 9, and 11. Tumors and TDLNs were dissected on day 14 after implantation (Figure 5A). Interestingly, tumor growth inhibition was not dose dependent; all doses exhibited comparable efficacy (Figure 5B). A slight drop in BW was observed in the mice injected with 4.5 μg of mcMEDI (Figure 5C). The dissected tumors showed no significant differences between the treated groups (Figure 5, D and E). The TDLNs from the groups administered higher doses showed significantly smaller sizes than those on the 0.5 μg regimen (Figure 5, D and F). Furthermore, significant cell loss was observed in 60%, 50%, and 0% of TDLNs after treatment with the 4.5 μg, 1.5 μg, and 0.5 μg regimens, respectively (Figure 5G and Supplemental Figure 11). When the mice were observed 60 days after surgery, the groups administered higher doses exhibited compromised systemic antitumor immunity, as evidenced by the compromised survival benefit (Figure 5H). Interestingly, the 4.5 μg dose completely abrogated the systemic antitumor effect of mcMEDI therapy despite significant primary tumor growth inhibition (Figure 5, B and H), implying the important role of TDLNs in systemic antitumor immunity (42). Given that the TLR 7/8 agonist also induces robust inflammation (43), its delivery to TDLNs at high doses may have induced nonresolving inflammation and irreversible damage to the tissue, with loss of functionality. Whereas a dose of 20 μg was used for conventional IT dosing in a previous preclinical study (4) and doses of 12 μg or 37 μg were used in a clinical trial (16), this regimen required a much lower dose (500 ng) to induce effective antitumor responses. Systemic administration of the same dose of mcMEDI induced significant BW loss, increased proinflammatory cytokines in the blood, and exhibited no primary tumor growth inhibition (Supplemental Figure 12). Taken together, these data suggest that the establishment of a local administration dose could play an essential role in inducing effective systemic antitumor immunity in in situ vaccination.

### Combination therapy with mcMEDI and immune checkpoint blockade for long-term antitumor immunity.

**Figure 6:**
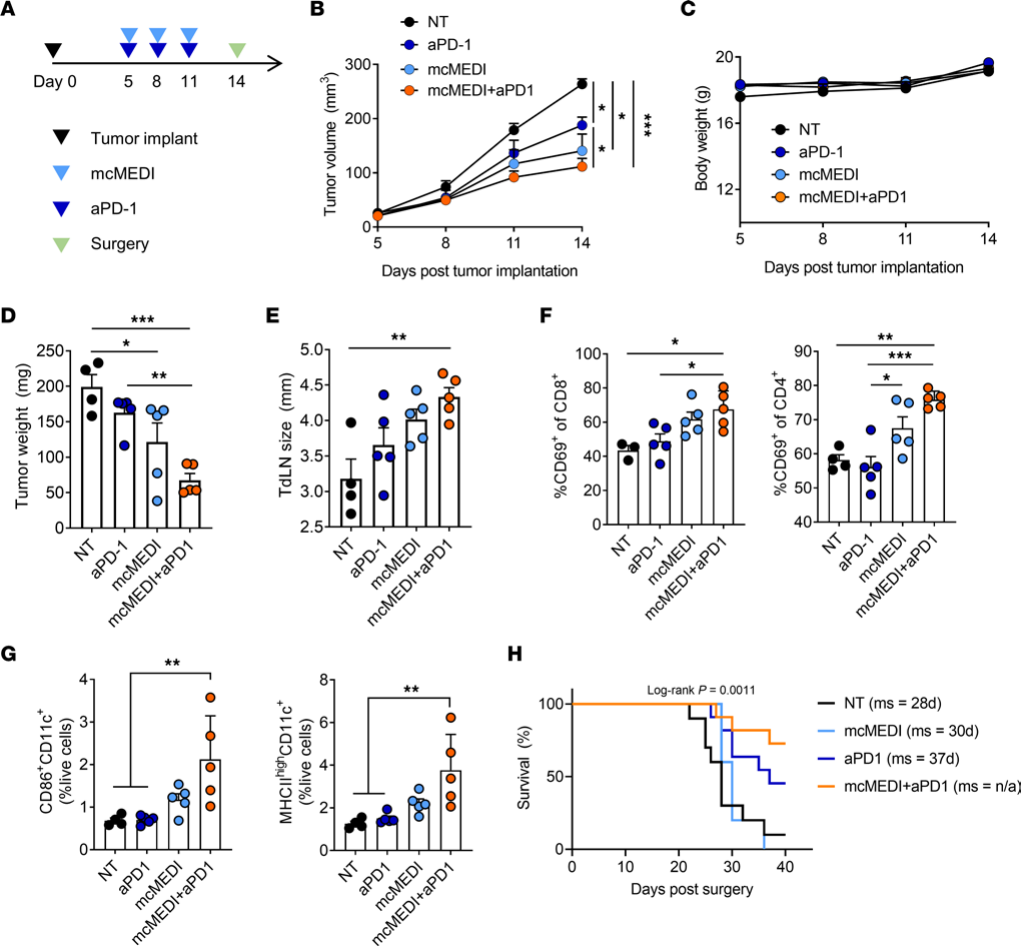
Combination therapy of mcMEDI and PD-1 blockade promotes long-term antitumor immunity as well as local immunomodulation of tumor and TDLN. (**A**) Schematic of combination treatment plan. BALB/c mice (*n =* 4–5 per group) were injected with 2×10^5^ 4T1-Luc mammary carcinoma cells into the fourth mammary fat pad (day 0). Anti–PD-1 Ab (200 μg) and mcMEDI (0.5 μg) were given i.p. and peritumorally on the indicated days, respectively. Tumors and TDLNs were dissected at day 14. (**B**) Primary tumor growth and (**C**) BW of mice during treatment. (**D**) Surgically removed tumor weight and (**E**) TDLN size at day 14. (**F** and **G**) Dissected tumors and TDLNs were digested into single cells, stained, and assessed by flow cytometry. (**F**) Percentages of activated cells (CD69^+^) in CD4^+^ T cells and CD8^+^ T cells in individual tumors are shown. (**G**) The frequency of DCs (CD11c^+^) and activated DCs (MHCII^+^, CD86^+^) in individual TDLNs are shown. (**H**) Kaplan-Meier survival curves of mice (*N =* 5–11 per group). Median survival days (ms) are listed. Data are representative of 2 independent experiments for B–G. Data presented as mean ± SEM. *P < 0.05, **P < 0.01, ***P < 0.001; 2-way RM ANOVA and Tukey’s multiple comparisons test for B and C, 1-way ANOVA and Tukey’s multiple comparisons test for D–G, and log-rank (Mantel-Cox) test for H. n/a, not achieved.

One of the challenges in developing new immuno-oncology is to integrate it rationally and effectively with conventional modalities, such as chemotherapy and immune checkpoint therapy. To assess the feasibility of combination therapy using mcMEDI, anti–PD-1 Abs were exploited and investigated whether the addition of mcMEDI improved their efficacy. After implantation of 4T1-Luc cells, anti–PD-1 Ab (200 μg) or mcMEDI (500 ng) was administered on the indicated days i.p. or peritumorally, respectively (Figure 6A). Monotherapy with anti–PD-1 Ab did not induce a significant decrease in tumor volume during treatment, implying moderate efficacy (Figure 6B). When anti–PD-1 Ab therapy was combined with mcMEDI therapy, significant growth inhibition was observed, suggesting that mcMEDI can improve primary tumor responses together with anti–PD-1 Ab. There was no significant difference in BW between the groups during treatment (Figure 6C). When tumors and TDLNs were dissected and analyzed at 14 days following implantation, the combination regimens induced significantly lower tumor weights and larger TDLN sizes (Figure 6, D and E, and Supplemental Figure 13, A and B). While monotherapy did not induce significant immune-related pathologic responses within the tumor and TDLN, the combination regimens exhibited significant immune responses (Figure 6, F and G). Moreover, the combination regimen exhibited a superior survival benefit compared with the other groups (Figure 6H). Although PD-1 blockade inhibits pre-existing CD8^+^ T cells from immune resistance against tumors (44), and since 4T1 is a “cold” tumor with an immune-excluded phenotype (45, 46), monotherapy with anti–PD-1 Ab was not sufficient for tumor-specific CD8^+^ T cells to infiltrate into the primary tumor and inhibit tumor growth during treatment. Nevertheless, circulating functional T cells with anti–PD-1 Abs could avoid immune resistance, remove remnant tumor cells after surgery and improve survival, as proven in clinical trials for various cancer types (47). In the combination regimen, mcMEDI produced a significant increase in the number of tumor-specific T cells via effective immunomodulation of TDLNs (Figure 3, F and G). Simultaneously, mcMEDI activated tumor-resident antigen-presenting cells to secret cytokines, such as IFN-α, IL-12, and IFN-γ (4), which can recruit T cells into the tumor tissue (Figure 3D and Figure 6F). Consequently, the combination regimen prolonged survival and inhibited primary tumor growth owing to the successful formation of systemic tumor-specific T cells in TDLNs and their infiltration into the primary tumor. Overall, these results showed that mcMEDI potently improved the efficacy of immune checkpoint therapy.

## Discussion

Currently, most local immunotherapies utilize IT administration. Although the potential for cancer treatment through TDLNs is well known (19), there have been few studies that actively utilize the immunomodulation of TDLNs in current modalities because of the lack of preclinical evidence and limitations of the delivery system. In this study, a practical strategy to exploit the antitumor functions of both tumors and TDLNs is presented, using PEGylated micelles as delivery vehicles.

The idea of targeting TDLNs for immune modulation is not new. Previous studies have shown that the TDLN-targeted delivery of immunotherapy can successfully induce immune-related pathological responses in tumors and TDLNs (20–24). However, there has been a lack of attempts to integrate this knowledge into current protocols. IT dosing is currently the mainstay for in situ vaccination because there is no good rationale to choose TDLNs over tumors as the primary target for delivery. In this study, instead of choosing one over the other, we showed that conventional dosing could lead to ineffective TDLN immunity modulation. PEGylated micelles could exploit effective antitumor functions of both tumors and TDLNs, demonstrating additive benefits for coimmunomodulation compared with conventional therapy. As MEDI9197 and other small molecule immune modulators are under clinical investigation (7, 16), this study provides a good rationale for developing formulations and drug delivery systems to reroute these modulators to promote antitumor immune responses.

In the dose escalation study, it was shown that the disruption of TDLNs characterized by significant cell loss could represent the dose-limited toxicity of the modality. The continuous accumulation of TDLNs indicates an inability to resolve acute inflammation, which leads to maladaptive immunity (39, 40). The magnitude and duration of immune responses are critical factors for controlling immune responses (38). A recent preclinical study demonstrated that excessive and cytotoxic dosing of STING agonists hampers tumor-specific T cell expansion and compromises durable antitumor immunity (41). These data suggest that dose optimization should be an integral part of local immunotherapy studies when considering systemic and local adverse events.

Although a change in tumor size is the standard measure of tumor response in conventional treatments, our data indicate that new guidelines may be required to accurately assess the efficacy of immunotherapy. In both tumor/TDLN immunomodulation and dose escalation studies, which represent neoadjuvant therapies, it was found that treatments that induced comparable tumor growth inhibition showed significantly different survival benefits. In the tumor/TDLN immunomodulation study of mcMEDI, the improved immunomodulatory activity in TDLNs could lead to a superior systemic antitumor response (Figure 3 and Figure 4). In the dose escalation study, while high doses (4.5 and 1.5 μg) induced tumor growth inhibition comparable with that at a low dose (0.5 μg), high doses resulted in compromised systemic antitumor immunity and survival (Figure 5). Although it could not be confirmed if the magnitude of TDLN activation exactly correlates with the magnitude of the systemic antitumor effect, it was deduced that a proper dosage is essential for successful systemic antitumor immunity, because cell loss in TDLNs or ineffective immunomodulation of TDLNs led to compromised survival benefits. Therefore, TDLNs play an important role in systemic and long-term antitumor immunity during in situ vaccination.

Scale-up is an important part of translating therapeutics from preclinical studies in small animal models to clinical use. In addition to their favorable physicochemical properties, PEGylated micelles have merits in that they are very easy to manufacture compared with other types of nanoparticles with similar properties (48). As PEGylated micelles are formed via the self-assembly of DSPE-PEG-2000 lipids, size control or homogenization processes are not required. For the incorporation of MEDI9197, it was simply dissolved and mixed with DSPE-PEG-2000 in an organic solvent, followed by drying and hydration. The optimization of the loading dose eliminated the requirement of a purification step. After drying, the film could be stored in a refrigerator and was available off the shelf. The hydration step required less than 30 minutes. A 5 mL glass vial was used for the preparation of mcMEDI incorporating 1 mg MEDI9197, which allowed 2000 injections of mcMEDI at a dose of 500 ng. As such, it is assumed that mcMEDI can be easily prepared and utilized for large animals or patients.

In conclusion, the results from this preclinical study provide a strong rationale for actively involving TDLNs in the course of local immunotherapies using micellar formulations for enhanced antitumor immune responses; thus, its clinical evaluation is warranted.

## Methods

### Study design.

The research objectives were to investigate whether coimmunomodulation of tumor and TDLNs during local immunotherapy can improve in situ vaccination and the feasibility of using PEGylated micelles for delivery of MEDI9197 to them. For assessment of local activity, tumor volume was measured during treatment. Physical examination of tumor and TDLNs was performed after dissection and they were subjected to flow cytometry analysis for immunological assessment. We mainly studied T cell and DC populations based on their acknowledged significant roles in antitumor immunity. For the assessment of systemic antitumor immunity, splenocytes were used for restimulation with tumor-specific antigen gp70_423-431_, whole body IVIS images were taken to observe distant metastasis, survival was observed, and tumor rechallenge in the distant site was performed. Dose escalation study was performed to establish optimal local dose and combination therapy with conventional modalities including chemotherapy and ICB therapy was performed to present the potential applicability of mcMEDI therapy with those treatments.

Mice were randomized after tumors were established to make tumor sizes similar between groups. The sample sizes were determined based on the results of pilot studies so that relevant statistical tests could reveal significant differences between experimental groups. For each experiment, mice numbers and statistical tests are described in the figure legends. Investigators were not blinded during evaluation of the in vivo experiments. Experiments were performed independently at least twice unless otherwise noted.

### Cell line and mouse.

The metastatic murine 4T1-Luc (Perkin Elmer) cell line was cultured in complete RPMI 1640 medium supplemented with 10% FBS and 1% penicillin/streptomycin. The MO5 (B16F10-OVA) cell line was cultured in complete DMEM supplemented with 10% FBS and 1% penicillin/streptomycin. The cells were tested for mycoplasma contamination and found to be negative. The MO5 cell line was gifted by H.K. Lee (Graduate School of Medical Science and Engineering, KAIST).

For all mice experiments, we used 7- to 10-week old female Balb/c mice or C57BL/6 mice that were purchased from Koatech (Gyeonggi-do).

### Micelles preparation.

DSPE-PEG-2000 (Avanti Polar lipids), lipophilic DiR (Thermo Fisher Scientific), and MEDI9197 (MedChem Express) were respectively dissolved in ethanol for stock preparation in round bottom glass vials. For micellar encapsulation, DSPE-PEG-2000 and each molecule were mixed at the desired molar ratios, dried under vacuum for film formation, and then stored at 4°C. The dry film was used within 1 month. For hydration, PBS was added and the samples were incubated in a shaking incubator at 37°C at 150 rpm for 20 minutes. For loading efficacy study, filtration through 0.22 μm syringe filters (Millipore) was performed to remove excessive MEDI9197 and its absorbance at 245 nm was measured for quantification. The hydrodynamic size and zeta potential of micelles were measured using dynamic light scattering (Zetasizer Nano ZS90).

### Biodistribution and pharmacokinetics.

Micelles incorporating DiR were prepared at a molar ratio 50:1 (DSPE-PEG-2000:DiR). For a conventional formulation, DiR was first dissolved in Tween 80 (40 mg/mL) and then ethanol (2.5 mg/mL) sequentially, which was diluted with PBS into a final concentration. For PT and IT injections, 50 μL and 10 μL of solutions were injected around the tumor and directly into the center of the tumor, respectively. The concentrations of micelles and DiR in the IT formulation were 5-fold higher than the PT formulation so the absolute injected doses were the same. For pharmacokinetics study, retro-orbital bleeding was performed to collect blood at different time points after injection. For biodistribution studies, mice were sacrificed at 96 hours after injection and the organs including the tumor, lymph nodes, liver, spleen, kidney, heart, lung, and muscle around the injection site were collected. NIR imaging system (Licor) was used to obtain ex vivo fluorescence images. For homogenization of solid tissues, Tissuelyer 2 (Qiagen) was used.

### In vivo tumor model and treatment.

For tumor implantation, BALB/c mice were injected with 2 × 10^5^ 4T1-Luc mammary carcinoma cells into the fourth mammary fat pad. For the tumor rechallenge study, the same number of cells were injected into the contralateral fourth mammary fat pad.

For mcMEDI administration, 50 μL of mcMEDI solution (0.5 μg MEDI9197) was injected locally into the peritumoral region. For conventional dosing, 10 μL of MEDI9197 was injected intratumorally into the center of the tumor mass. MEDI9197 was first dissolved in Tween 80 (40 mg/mL) and then ethanol (2.5 mg/mL) sequentially, which was diluted into a final concentration of 50 μg/mL. For anti–PD-1 dosing, 200 μg of anti–PD-1 Abs (Bio X Cell, RMP1-14) was injected i.p.

For the imaging of 4T1-Luc cells in mice, 100 μL of Vivoglo luciferin substrate solution (30 mg/mL; Promega) was i.p. injected into mice 10 minutes before imaging. The luminescence image was obtained using IVIS by detecting luminescence intensity without any light excitation.

For survival data, euthanized mice and naturally dead mice were recorded. Mice were euthanized in either of the 2 following cases; a) when the tumor exceeds 10% of normal weight (diameter over 15 mm, approximately), or b) when there is a decrease in BW by at least 20% of normal weight.

### FACS analysis.

Tissues were collected in RPMI media containing 200 μg/mL Liberase TM (Roche, 5401119001) and 50 U/mL DNase I (Thermo Fisher Scientific), minced into small pieces, and then incubated for 90 minutes at 37°C with agitation. Then, the cells were passed through a 70 μm cell strainer and washed by centrifugation at 350 *g* for 5 minutes. The cells were resuspended in FACS buffer (5% FBS in PBS) after centrifugation for surface staining. For surface staining, the cells were incubated with surface marker Abs for 30 minutes at 4°C. The cells were washed once with FACS buffer and resuspended in FACS buffer for flow cytometric analysis. For live/dead staining, cells were stained with zombie aqua (BioLegend) or propidium iodide (Thermo Fisher Scientific) according to recommended protocols.

For PBMC analysis, the whole blood samples were incubated with surface marker Abs for 20 minutes at room temperature. Red blood cells were lysed in 1X RBC Lysis Buffer (BioLegend) at room temperature. The blood samples were then centrifuged at 350 *g* for 5 minutes and then resuspended in FACS buffer for analysis. Cells were acquired on BD LSRFortessa (Becton Dickinson). To measure blood proinflammatory cytokines, treatment was given twice every other day and the blood was collected 2 days after the last injection. The blood concentration of TNF-α and IFN-γ were measured using ELISA.

The following mAbs were used for flow cytometry: CD16/32 (Fc blocker) (BioLegend, 101302; 93); CD45-APCeF780 (Invitrogen, 47-0451; 30-F11); CD3-FITC (Invitrogen, 11-0032; 17A2) or APC-Cy7 (Tonbo, 25-0032; 17A2); CD8α-APC (Invitrogen, 17-0081; 53-6.7) or PerCP Cy5.5 (Tonbo, 65-0081; 53-6.7); CD4-eF450 (Invitrogen, 48-0042; RM4-5); CD4-FITC (Tonbo, 35-0042; RM4-5) or -PE (Tonbo, 50-0041; RM4-5); CD11c-PE (Tonbo, 50-0114; N418); CD86-FITC (Tonbo, 35-0862; B7-2); MHCII-eF450 (Invitrogen, 48-5321; I-A/I-E) or -APC (Tonbo, 20-5321; I-A/I-E); CXCR3-APC (BioLegend, 126511; CXCR3-173) or –PerCP Cy5.5 (BioLegend, 126514; CXCR3-173); CD69-FITC (Tonbo, 35-0691; H1.2F3); and CD25-PE (Invitrogen, 12-0251; PC61.5).

### Splenocytes restimulation.

To assess systemic antitumor immunity after treatment, mice were sacrificed 4 days after surgery. The spleen was collected in complete RPMI media, gently minced, and filtered through a 70 μm cell strainer using a syringe plunger. Splenocytes were centrifuged at 350 *g* for 10 minutes at 4°C. The cells were resuspended in ACK lysing buffer (Thermo Fisher Scientific) for lysing of red blood cells. After resuspension, cells were counted, diluted, and seeded onto a 48-well plate (SPL Life Science) at a cell density of 5 × 10^6^ cells/mL. For restimulation, 100 μg of tumor-specific antigen gp70_423-431_ (Peptron), a tumor-specific immunodominant peptide, was treated. Cells were incubated for 96 hours and the supernatant was collected for measurement of IFN-γ secretion using ELISA.

### Statistics.

A *P* value less than 0.05 was considered significant. Data were analyzed using the unpaired 2-tailed Student’s *t* test for 2 groups and 1-way ANOVA or 2-way RM ANOVA for 3 or more groups followed by Tukey’s multiple comparisons test using Prism 8.4 (GraphPad Software). Kaplan-Meier survival curves were compared with the log-rank test (Mantel-Cox).

### Study approval.

All animal procedures were approved by the Animal Care and Use committees at KAIST.

## Author contributions

MJ and HK designed the study, conducted the experiments, acquired the data, and wrote the manuscript. JY conducted the experiments. DHK and JHP reviewed the analysis and wrote the manuscript. HK and JHP supervised the study. All authors contributed to manuscript revisions. MJ and HK are co–first authors. As MJ contributed the most during the revision process, MJ came first in the order of authorship.

## Supplementary Material

Supplemental data

## Version 1 05/17/2022

In-Press Preview

## Version 2 06/22/2022

Electronic publication
