# Supplementary material for: Coimmunomodulation of tumor and tumor-draining lymph nodes during in situ vaccination promotes antitumor immunity
Source: JCI Insight. 2022 Jun 22;7(12):e146608. doi: 10.1172/jci.insight.146608 (PMC9309043; doi:10.1172/jci.insight.146608)
Supplement: Supplemental data [file jciinsight-7-146608-s058.pdf]

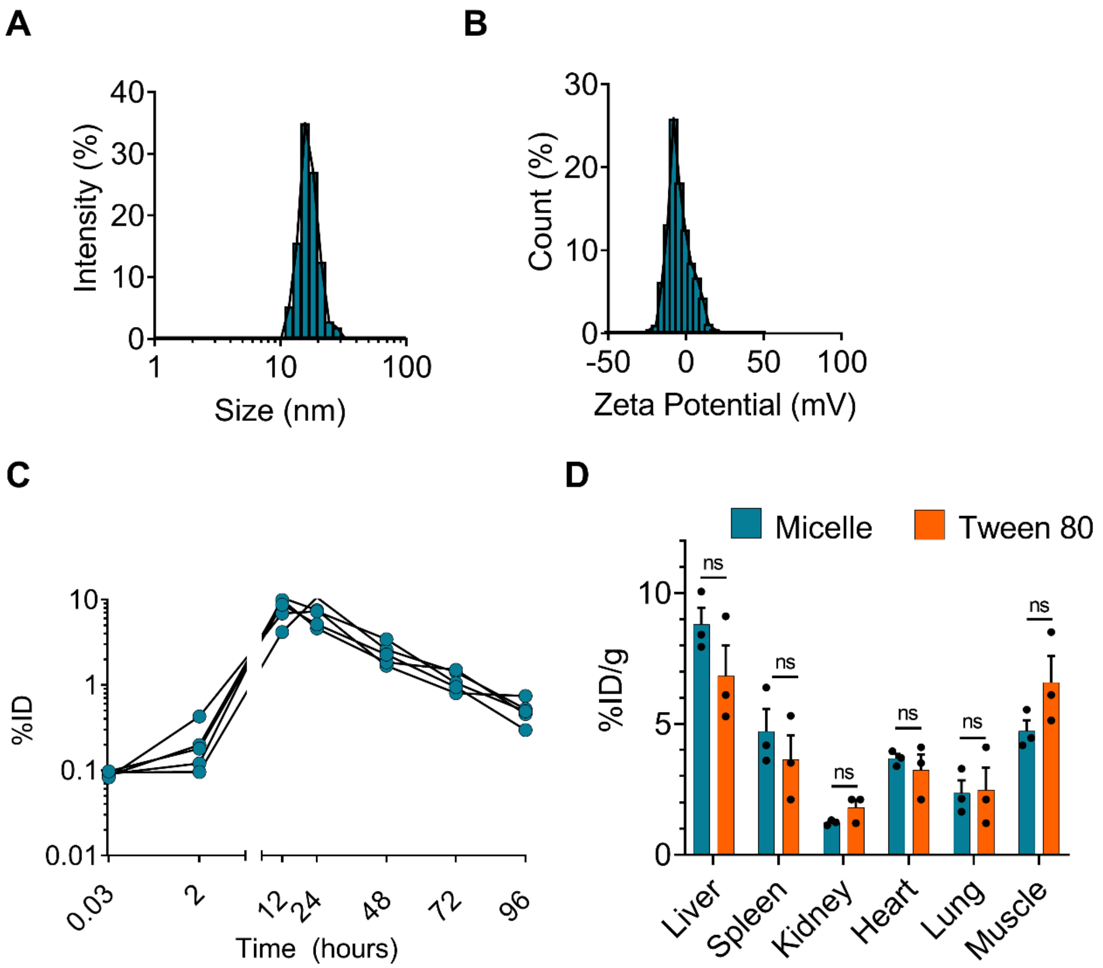

Supplemental Figure 1. Micelles characterization and pharmacokinetics. (**A**, **B**) The distribution profiles of hydrodynamic diameter (**A**) and zeta potential (**B**) were shown. (**C**) Pharmacokinetics of micelles. 50 µl of micelles incorporating DiR were injected peritumorally. The percent injected dose at indicated timepoints in blood (n = 5). (**D**) Biodistribution in various organs at 96 hours after the injection of micelles incorporating DiR (50 µl, peritumoral) or DiR solution (10 µl, intratumoral). Micelles incorporating DiR were prepared at a molar ratio 50:1 (DSPE-PEG-2000:DiR). For DiR solution, DiR was firstly dissolved in Tween 80 (40 mg/ml) and then ethanol (2.5 mg/ml) sequentially, which was diluted with PBS into a final concentration. (n = 3). Data present mean ± SEM. ns (not significant). Multiple t-tests were performed.

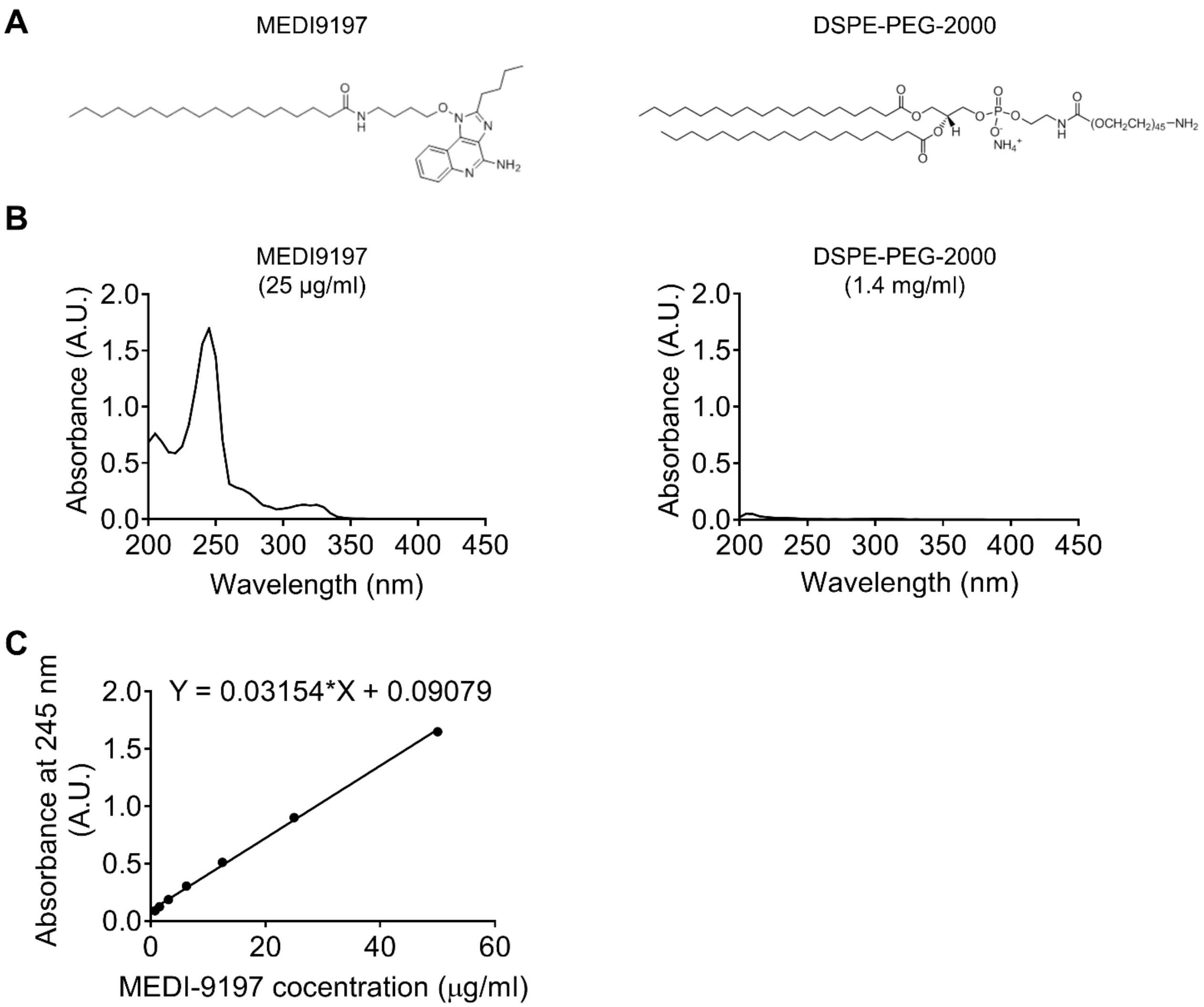

Supplemental Figure 2. Molecular structures and UV spectrum of MEDI9197 and DSPE-PEG-2000. (**A-B**) Molecular structures (**A**) and UV absorption spectrum (**B**) of MEDI9197 and DSPE-PEG-2000. The absorbance spectrum was measured using ethanol as solvent at the indicated concentrations. (**C**) Standard curve of MEDI9197 concentration versus absorbance at 245 nm. The concentration of MEDI9197 was determined using the equation Y=0.03154*X+0.09079 with an R^2 value of 0.9987, where Y is absorbance and X is concentration. The experiment was performed once.

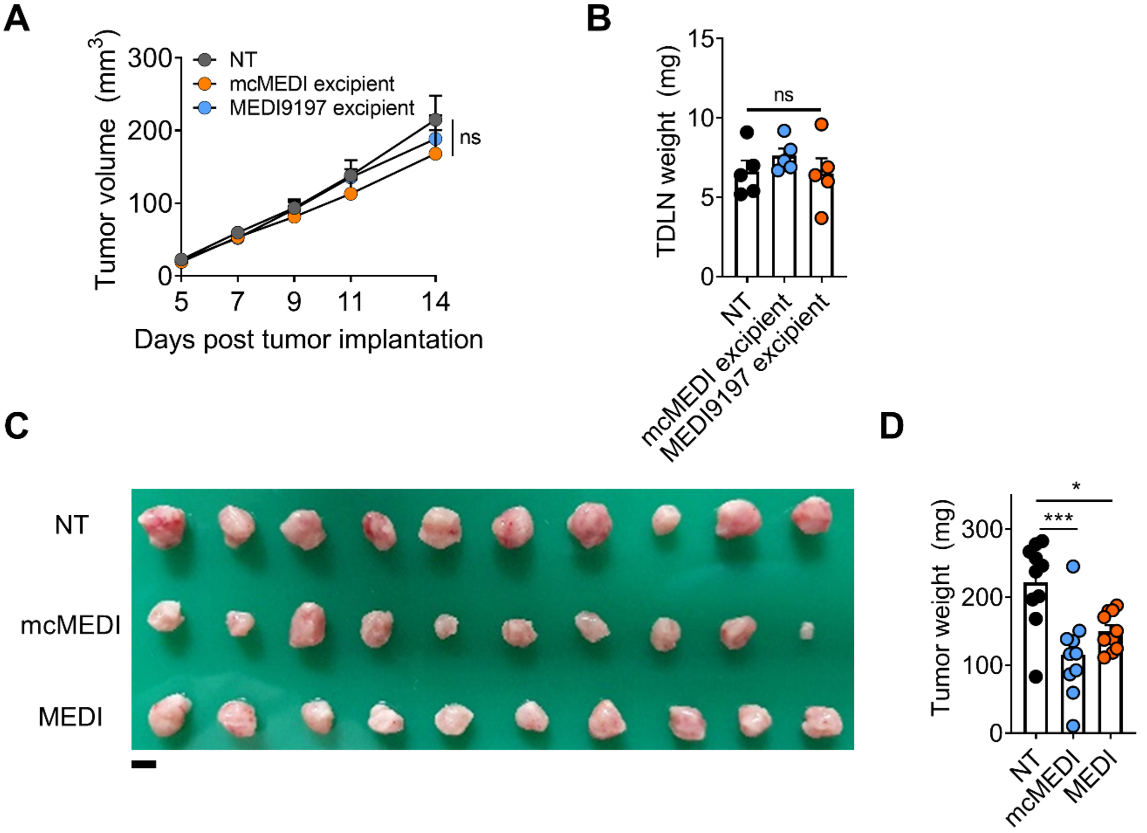

Supplemental Figure 3. In situ vaccination with mcMEDI effectively inhibits primary tumor growth. (**A**, **B**) BALB/c mice were injected with 2×10^5 4T1-Luc mammary carcinoma cells into the fourth mammary fat pad. When the tumor size reached approximately 50 mm^3, 50 µl of micelle excipient (micelle solution without MEDI9197) and 10 µl of MEDI9197 excipient (PBS containing 0.125% Tween 80 and 2% ethanol) was injected peritumorally and intratumorally, respectively (N = 5 per group). The injections were performed twice every other day as described in Figure 3. Primary tumor growth (**A**) and weight of TDLN (**B**) removed from mice on day 14. This experiment was performed once. (**C**, **D**) When the tumor size reached approximately 50 mm^3, treatment groups were given either micellar MEDI9197 (mcMEDI) peritumorally (PT) or free MEDI9197 intratumorally (IT) at a dose of 0.5 µg per injection. Injections were performed twice every other day as described in Figure 3. Ex vivo images of the primary tumor surgically removed from mice on day 14 (**C**) and their weight (**D**). The scale bar indicates 5 mm. Data present mean ± SEM. ns (not significant). *P<0.05, ***P<0.001. One-way ANOVA and Tukey's multiple comparison test was performed.

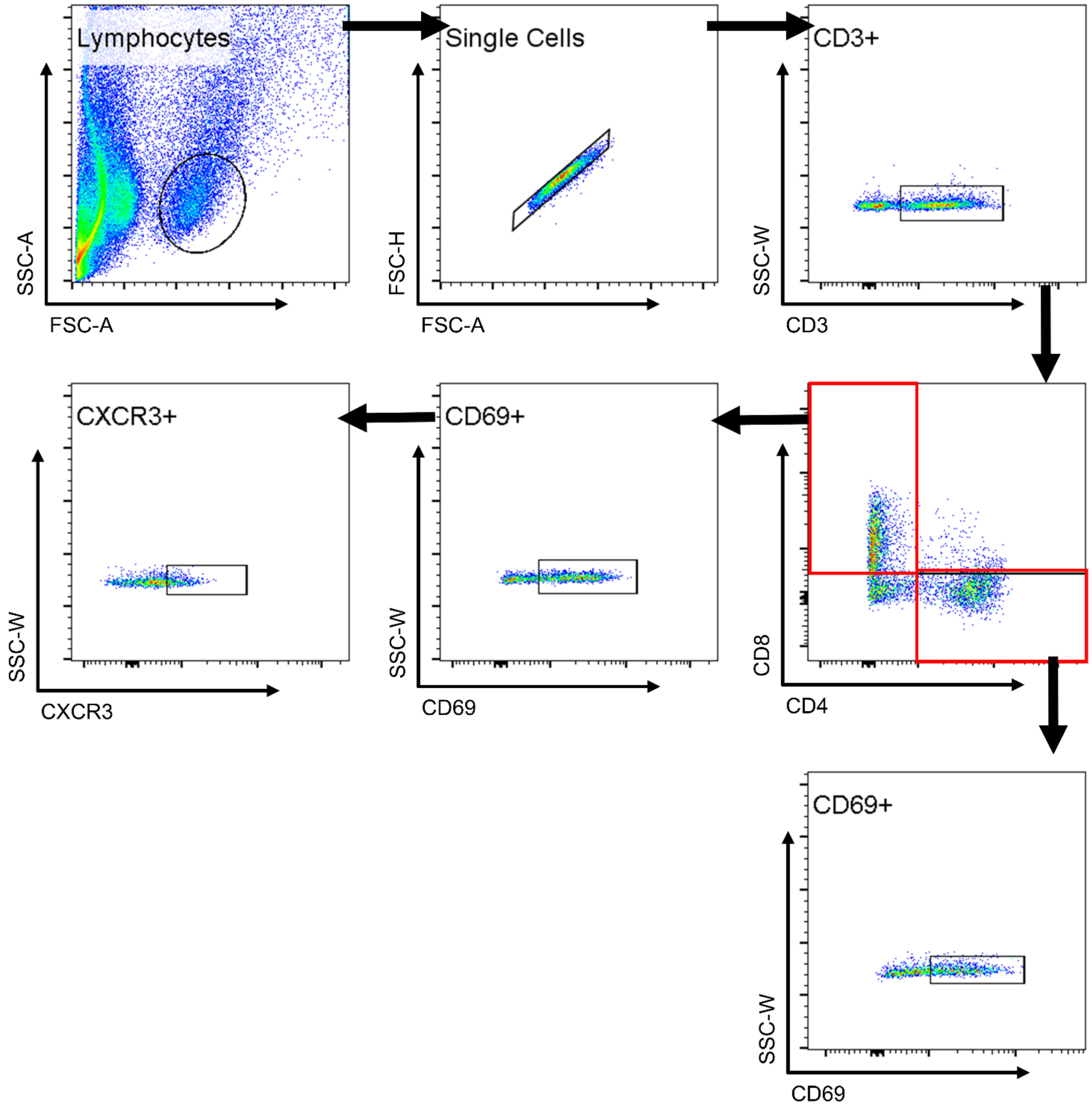

Supplemental Figure 4. Gating strategy for tumor-infiltrated lymphocytes. Lymphocyte populations were firstly selected based on forward and side scatter (FSC-A and SSC-A). The cells were plotted in FSC-A and FSC-H to gate single ells and exclude doublets. From the single cell gate, T cells were defined as $CD3^+$ cells. T cells were further divided into $CD4^+$ and $CD8^+$ populations. Activation and memory phenotypes were examined with CD69 and CXCR3 surface markers, respectively.

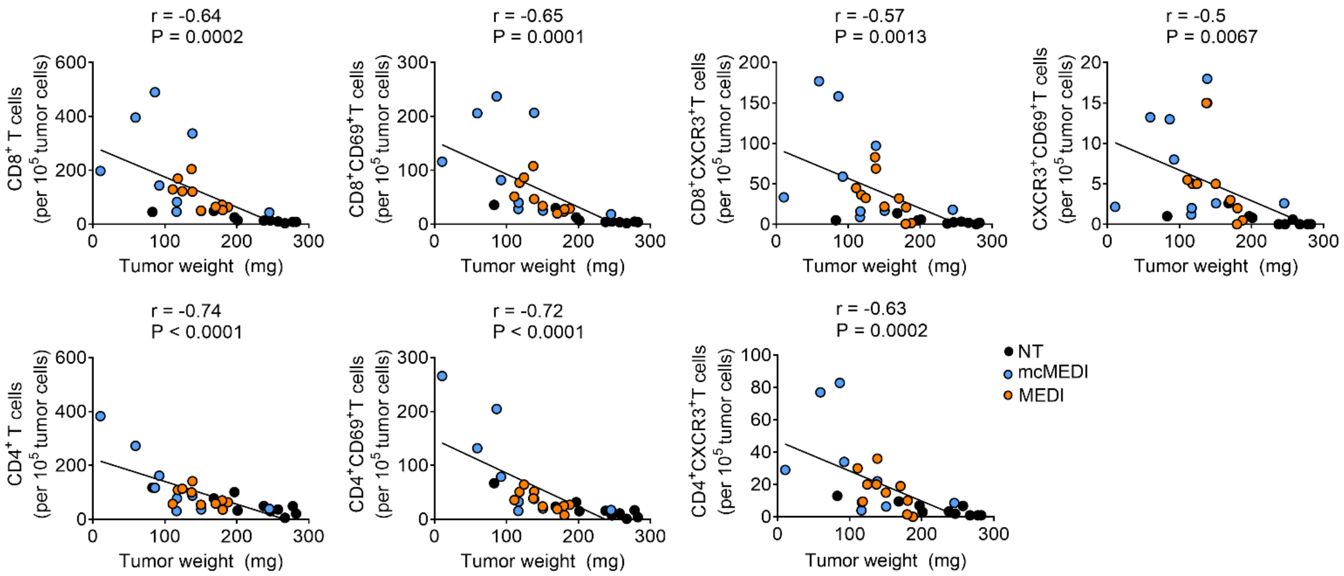

Supplemental Figure 5. Tumor weight exhibits negative correlation with the density of tumor-infiltrated lymphocytes. Pearson correlation analysis was performed between tumor weight and the density of tumor infiltrated lymphocytes (N = 10 per group).

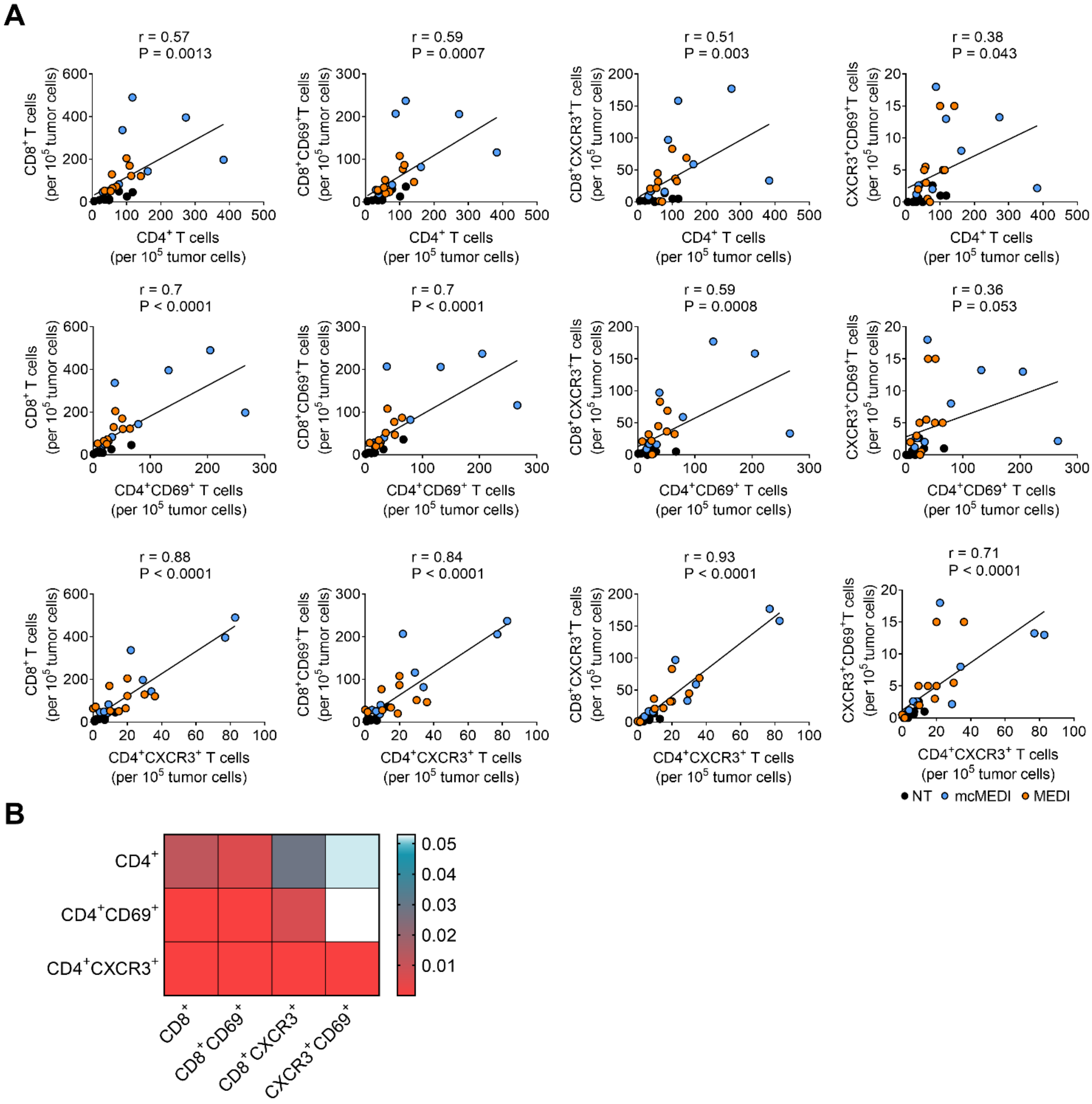

Supplemental Figure 6. The density of tumor-infiltrated CD4^{+} and CD8^{+} T cells exhibit positive correlation. (**A**) Pearson correlation analysis was performed between various subsets of CD4+ and CD8+ T cells (N = 10 per group). (**B**) The heat map shows strong positive correlation between Th1 CD4^{+} T cells and CD8^{+} T cells and their activated phenotypes.

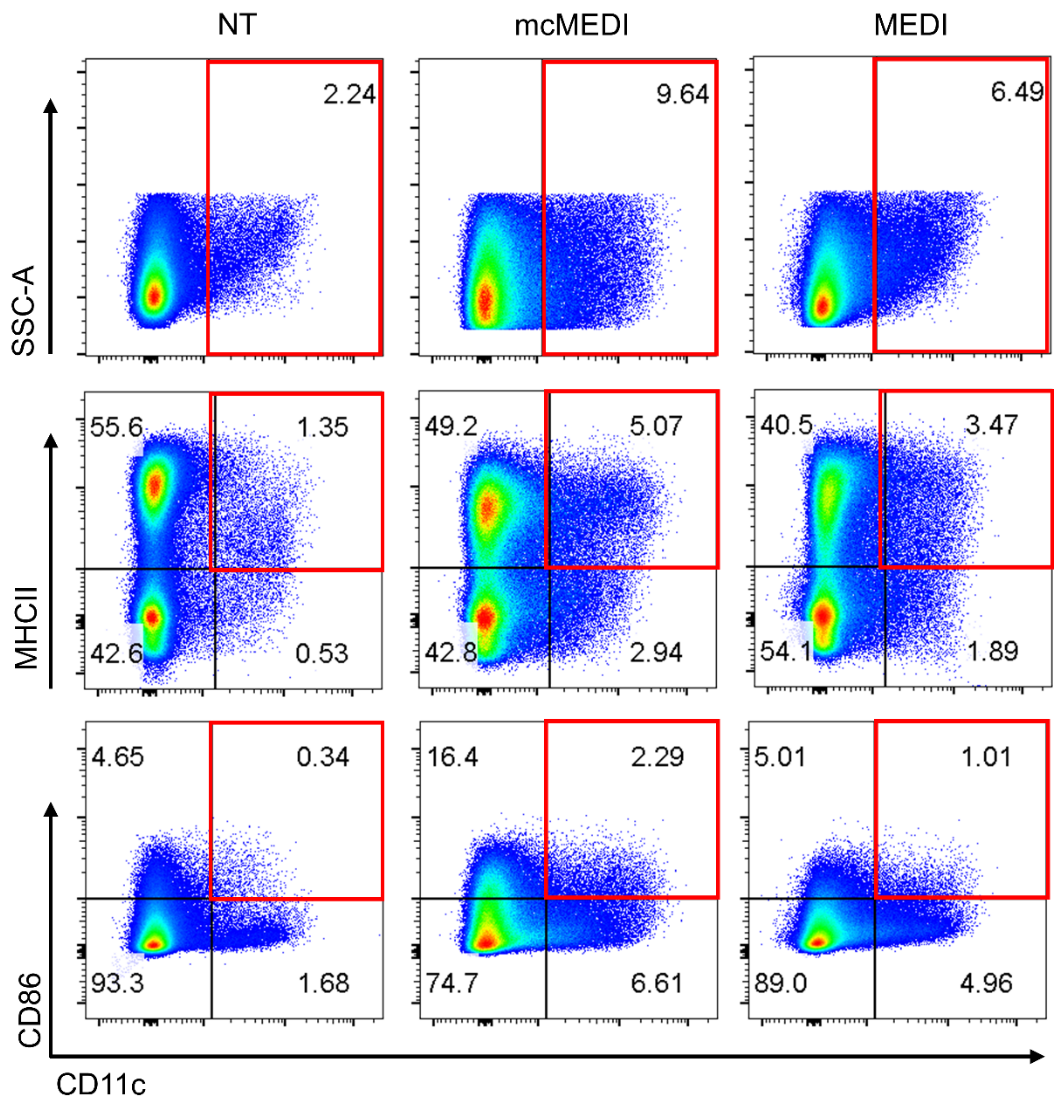

Supplemental Figure 7. mcMEDI effectively activates innate immunity in TDLN. The excised TDLNs were digested to isolate single cells and then the cells were stained and assessed by flow cytometry. Dendritic cells (DC) ($CD11c^+$), and activated DCs ($MHCII^+$ or $CD86^+$) in individual TDLNs are analyzed.

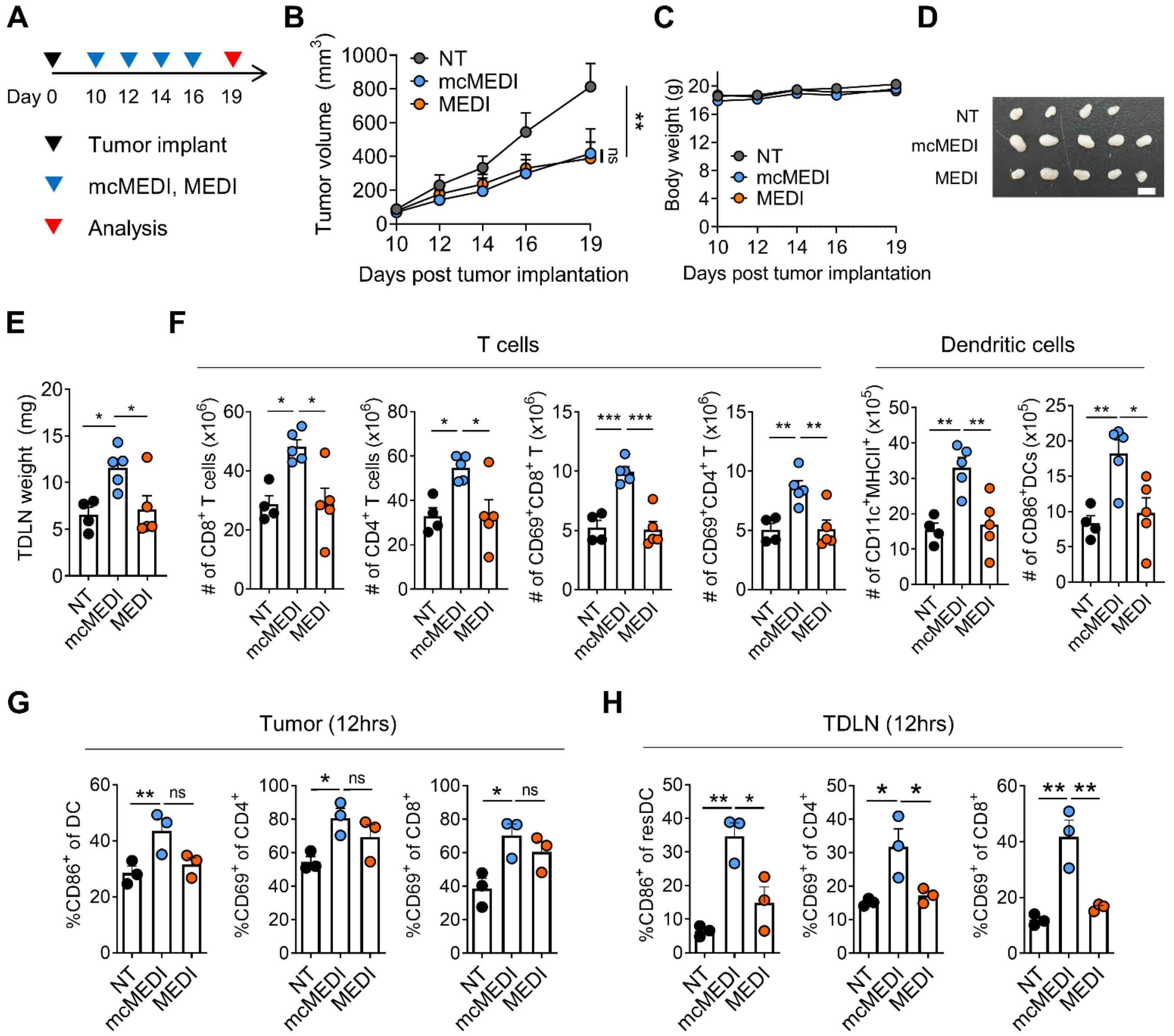

Supplemental Figure 8. mcMEDI therapy induces effective local immune activity within primary tumor and TDLN in mouse melanoma model. (**A**) Schematic of treatment plan. C57BL/6 mice (N = 4-5 per group) were subcutaneously injected with 1×10^6 MO5 (B16F10-OVA) melanoma cells (day 0). 50 µl of mcMEDI was injected into the peritumoral region approximately 5 mm away from the tumor while 10 µl of MEDI9197 was injected directly into the center of the tumor. Both treatment groups were given MEDI9197 at a dose of 0.5 µg per injection at the indicated days. The no treatment (NT) group did not receive MEDI9197. (**B**) Primary tumor growth and (**C**) body weight of mice during therapy. Ex vivo image (**D**) and the weight (**E**) of TDLN at day 19. The scale bars indicate 5 mm. (**F**) The TDLNs were

digested to isolate single cells, stained and assessed by flow cytometry. The number of T cells, activated T cells, dendritic cells ($CD11c^{+}MHCII^{+}$), and activated DCs ($CD86^{+}$) in individual TDLNs are shown. **(G-H)** C57BL/6 mice (N = 3 per group) were subcutaneously injected with 1×10^{6} MO5 (B16F10-OVA) melanoma cells (day 0). 0.5 μg of mcMEDI (P.T.) or MEDI9197 (I.T.) was injected once on day 10. Dissected tumors and TDLNs at 12 h after injection were digested into single cells, stained and assessed by flow cytometry. (**G**) Percentages of activated cells in dendritic cells ($CD86^{+}$) or in T cells ($CD69^{+}$) in tumor tissue. (**H**) Percentages of activated cells in resident dendritic cells ($CD86^{+}$) or T cells ($CD69^{+}$) in TDLNs. Data is a representative of two independent experiments. Data present mean ± SEM. n.s. (not significant). *P<0.05, **P<0.01, ***P<0.001, two-way ANOVA and Tukey's multiple comparison test for B and C, one-way ANOVA and Tukey's multiple comparison test for E-H.

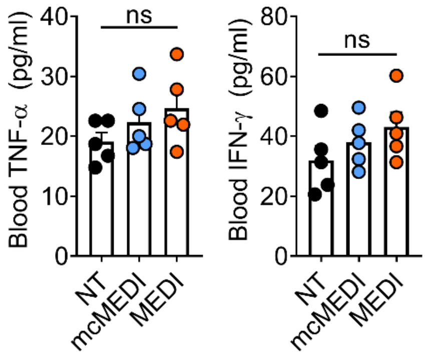

Supplemental Figure 9. Blood concentration of proinflammatory cytokines during treatment. Treatment groups were given MEDI9197 at a dose of 0.5 µg per injection (N = 5 per group). Injection was performed twice every other day and the blood was collected 2 days after the last injection. The blood concentration of TNF-α and IFN-γ were analyzed using ELISA, respectively. Data present mean ± SEM. n.s (not significant). One-way ANOVA and Tukey's multiple comparison test was performed.

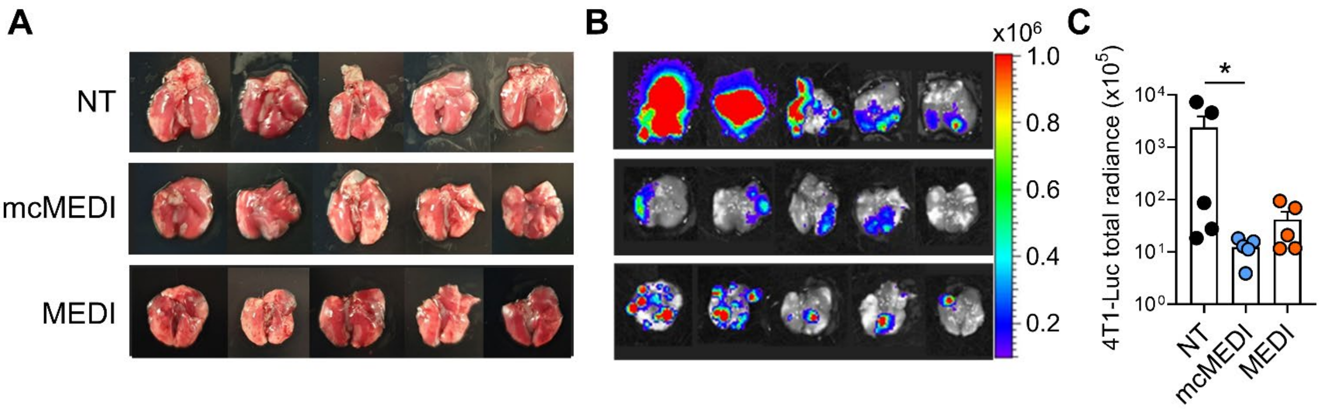

Supplemental Figure 10. Anti-metastatic effect of mcMEDI in 4T1-Luc lung metastasis model. Treatment groups were given either mcMEDI peritumorally or free MEDI9197 intratumorally at a dose of 0.5 µg per injection (N = 5 per group). Injection was performed twice every other day (day 5, 7, 9, and 11). The primary tumor, TDLN, and mammary fat tissue were surgically removed without the removal of tumor-overlying skin. The lung was excised at 21 days after surgery. (**A**) The images of lung tissue excised from mice at 21 days after surgery. The excised lung was imaged via IVIS (**B**) and the total luminescence radiance of each lung was measured (**C**). The color bars indicate luminescence radiance (p/s/cm^2/sr). Kruskal-Wallis test was used for C. Data present mean ± SEM.

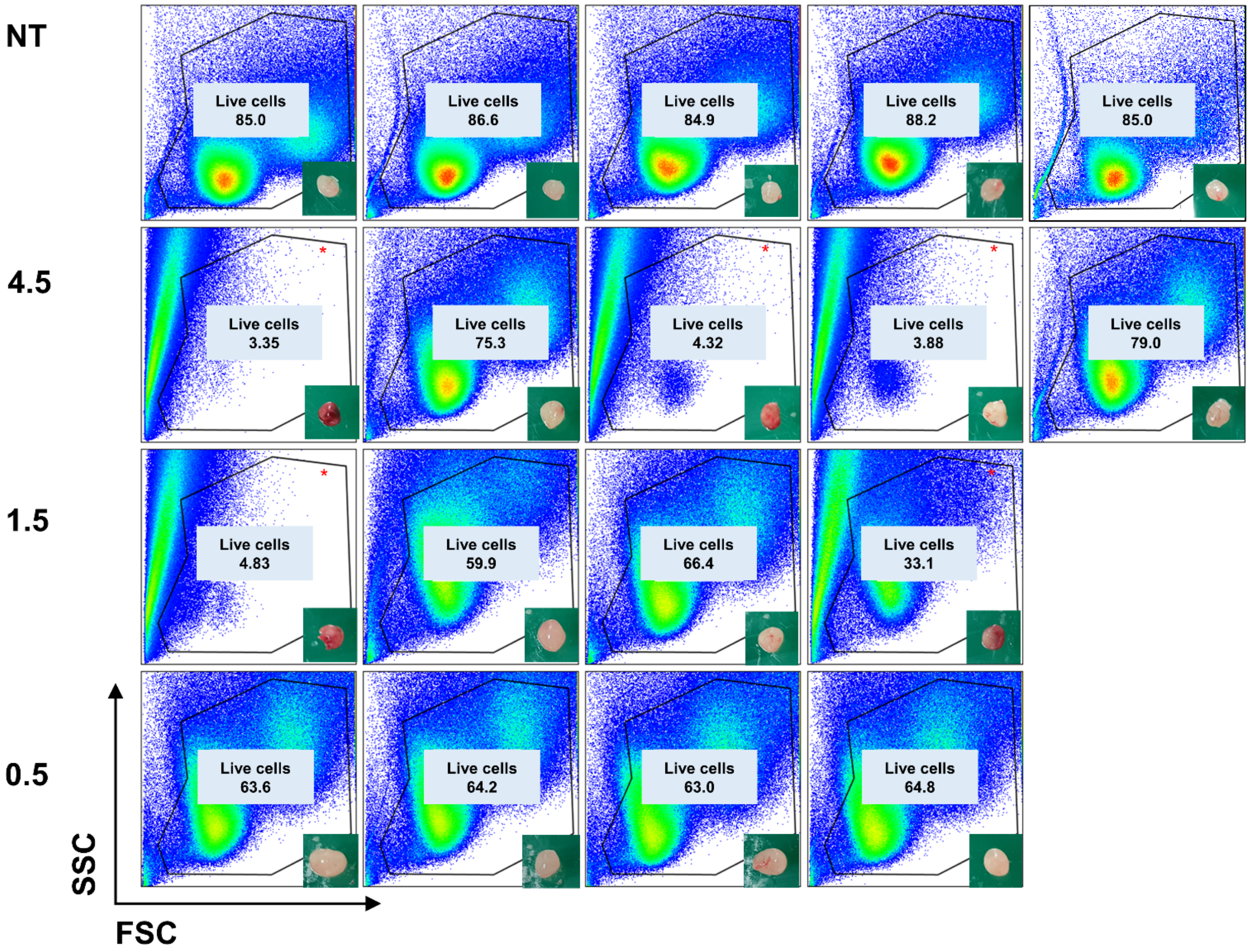

Supplemental Figure 11. TDLN cell loss in mice treated with high doses of mcMEDI. The excised TDLNs were digested to isolate single cells and then assessed by flow cytometry. Live cells were gated based on FSC and SSC and the pictures of the analyzed TDLN were embedded on the right bottom of each FACS plot.

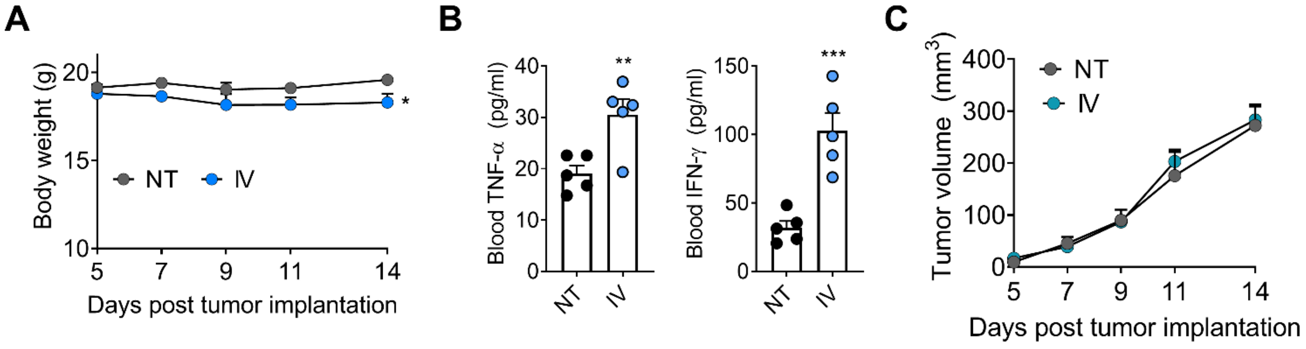

Supplemental Figure 12. mcMEDI is not therapeutically effective when intravenously administered. BALB/c mice (N = 5 per group) were injected with 2×10^5 4T1-Luc mammary carcinoma cells into the fourth mammary fat pad (day 0). MEDI9197 treatment groups were given mcMEDI intravenously (IV) at a dose of 0.5 µg per injection at 5, 7, 9, and 11 days post-implantation. (**A**) Body weight during therapy. (**B**) Blood proinflammatory cytokines concentration on day 9. (**C**) Tumor growth during therapy. The experiment was performed once. Data present mean ± SEM. *P<0.05, **P<0.01, ***P<0.001, multiple t-test for A, C, unpaired two-tailed t-test for B.

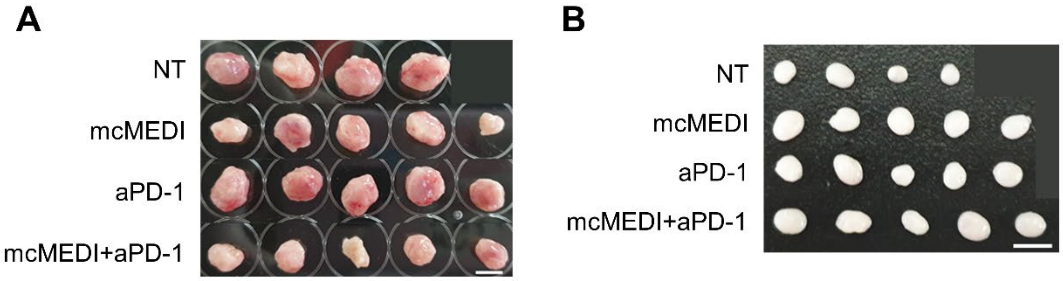

Supplemental Figure 13. Combination with mcMEDI and anti PD-1 antibody shows superior anti-tumor effect and immunomodulation of TDLN. Treatment groups were given mcMEDI (0.5 µg) peritumorally or anti PD-1 antibody (aPD-1; 200 µg) intraperitoneally (N = 4-5 per group). Injection was performed on day 5, 8, and 11. Ex vivo images of primary tumor (**A**) and tumor draining lymph nodes (**B**) dissected at day 14. The scale bar indicates 50 mm (**A**) and 5 mm (**B**), respectively.

Supplemental Table 1. Characterization of micelles used in the study. Hydrodynamic size, polydispersity index (PDI), and zeta potential of DiR and MEDI9197-loaded micelles were measured by dynamic light scattering. Data present mean ± SEM. (n = 3).

Cargo	Size	PDI	Zeta potential
**DiR**	17.1 ± 2.0 nm	0.04 ± 0.03	-3.5 ± 7.0 mV
**MEDI9197**	14.3 ± 1.7 nm	0.07 ± 0.06	-1.8 ± 0.5 mV
